# Thermal Characterization of Rarefied Flows in Rhombic Microchannels

**DOI:** 10.3390/mi14122222

**Published:** 2023-12-10

**Authors:** Pamela Vocale, Gian Luca Morini

**Affiliations:** 1 Department of Engineering and Architecture, University of Parma, Parco Area delle Scienze 181/A, 43124 Parma, Italy; 2 Department of Industrial Engineering, Alma Mater Studiorum Università di Bologna, Viale Risorgimento 3, 40135 Bologna, Italy; gianluca.morini3@unibo.it

**Keywords:** micro flow, rarefaction effects, viscous dissipation, noncircular cross-sections

## Abstract

This work aimed to numerically investigate the dynamic and thermal behavior of a fully developed, laminar, gaseous flow in a microchannel featuring a rhombic cross-section. Due to new fabrication techniques, microducts with rhombic cross-sections have recently received more attention. The momentum and energy balance equations were solved by using a commercial CDF code and assuming the slip and the H2 boundary conditions. The temperature jump between the wall and the adjacent fluid was also taken into account. The accuracy of the numerical results was checked by using the data available in the literature in terms of velocity profiles in the slip flow regime and the Nusselt number in the continuum flow regime. To also investigate the geometry effects on the fluid behavior, several values of the side angle of the rhombus were considered. The numerical results revealed that the rarefaction degree and geometrical properties significantly affected the Nusselt number.

## 1. Introduction

Research on more efficient components is one of the main scientific and technological challenges of recent decades. Among the passive techniques for heat transfer enhancement developed and studied in the literature, device miniaturization plays an important role.

When the characteristic length of the channels decreases, rarefaction effects increase, and different flow regimes (slip flow, transition, and free molecular regimes) should be considered [1].

Rarefied gas flows in circular microchannels have been deeply investigated [2,3,4,5,6,7,8,9,10,11,12,13,14,15]. Particularly, Ameel et al. [5] presented an analytical solution to the convective heat transfer of gaseous flow, in the slip regime, by considering a constant heat flux. Their results reveal that the Nusselt number decreases with increasing Knudsen number. Hong and Asako [12] numerically investigated the performance of slip flows in microtubes characterized by a hydraulic diameter ranging between 10 to 100 μm and subjected to constant heat flux. They concluded that rarefaction was dominant for low values of the Reynolds number.

The impact of rarefaction in microchannels with rectangular cross-sections has also been investigated by many researchers [16,17,18,19,20,21,22,23,24,25,26,27]. Particularly, Yu and Ameel [19] presented an analytical investigation of slip flows in microchannels featuring a rectangular cross-section by assuming a uniform wall heat flux. They found that the influence of the rarefaction effect on the convective heat transfer depended on the aspect ratio and on the rarefaction degree. Kuddusi and Çetegen [24] numerically analyzed the laminar forced convection in the slip regime by assuming a constant and uniform wall heat flux. Their results confirm that, also for rectangular microchannels, the higher the rarefaction degree, the lower convective the heat transfer coefficient.

The thermal performance of rarefied flows in trapezoidal microchannels was assessed in [28,29,30,31]. Particularly, the influence of rarefaction effects under wall heat flux boundary condition were numerically investigated by Cao et al. [28] and Kuddusi and Çetegen [29]. Their numerical findings highlighted the impact of rarefaction and aspect ratio on heat transfer.

The evaluation of the rarefaction effects in different cross-section geometries was carried out by Zhu et al. [32] and by Vocale et al. [33]. More specifically, a theoretical analysis of slip flow heat transfer in triangular microchannels with constant wall heat flux is presented in [32]; a numerical investigation of the impact of rarefaction in elliptical microchannels with constant wall heat flux is presented in [33]. The results presented in [32,33] revealed that, also for triangular and elliptic microchannels, the influence of rarefaction depended on the aspect ratio.

Although microchannels with circular, rectangular, and trapezoidal cross-sections have been fabricated and investigated for many years, microducts featuring rhombic cross-sections have recently received more attention because of new fabrication methods. Rhombic microchannels can be fabricated by binding two triangular microchannels on top of each other or can be obtained from the etching of rectangular microchannels when the side angles are not 90° [34].

The dynamic behavior of fully developed, laminar, single-phase flow in rhombic microchannels has been investigated by many authors who have proposed approximate or analytical solutions for several channel cross-sections [35,36,37,38,39,40]. In particular, Bahrami et al. [35] presented an approximate solution for predicting the pressure drop in singly connected microchannels with different cross-sections by considering the square root of the cross-sectional area as the characteristic length. Tamayol and Bahrami [36] proposed analytical solutions in the form of trigonometric series to predict velocity profiles and pressure drops for various geometries with different shapes. Compact solutions, obtained in the form of infinite series, were presented by Shahsavari et al. [37] to evaluate velocity and temperature profiles in a wide range of geometries. 

More recently, Saha et al. [38] presented a numerical investigation of the thermal behavior of laminar flows in rhombic microchannels by assuming water as a working fluid. 

On the other hand, slip flow in rhombic microchannels was investigated only by a few researchers, although rarefaction effects may significantly affect the performance of miniaturized devices.

Closed form solutions for evaluating the pressure drop for fully developed flow within the slip flow regime through microchannels featuring polygonal, rectangular, and rhombic cross sections were developed by Tamayol and Hooman [39]. 

Shams et al. [40] numerically investigated the influence of Reynolds and Knudsen numbers on Poiseuille and Nusselt numbers in rhombic microchannels by considering the T boundary condition (i.e., constant wall temperature). Moreover, the effects of the aspect ratio were studied. Their findings revealed that the aspect ratio and the Knudsen number significantly affect Poiseuille and Nusselt numbers.

Baghani et al. [41] conducted a theoretical study to develop compact solutions obtained in the form of infinite series, which enabled the evaluation of velocity and temperature profiles in polygonal, trapezoidal, rhombic, and elliptic microchannels. The solutions presented in [41] were obtained by considering the slip flow regime and the H1 boundary condition [42]. 

From the literature review, it is evident that slip flow forced convection has been investigated only by assuming the T and the H1 boundary conditions. Hence, there is a need to investigate slip flow forced convection in an H2 boundary condition (i.e., constant wall heat flux [42]). 

In this work, a numerical investigation of the dynamic and thermal behavior of a fully developed, laminar, gaseous flow through a rhombic microchannel is presented. The governing equations with the slip and the H2 boundary conditions were solved by using a commercial CDF code. The temperature jump between the wall and the adjacent fluid was also accounted for. 

The accuracy of the numerical results was checked by comparing the numerical velocity profiles to the distributions obtained by applying closed-form solutions to the fully developed slip flow available in the literature [39]. Moreover, for the no-slip flow, the numerical values of the Nusselt number were compared to the data available in the literature [42].

The main goal of the present study is to assess the performance of the rhombic microchannels, which can be fabricated thanks to the new microfabrication technique, to understand if this kind of geometry enables to achieving good thermohydraulic performance. The geometric design of the microchannel plays an important role in enhancing the thermal performance of the microchannel heat sinks or microchannel heat exchangers [43].

The novelty of this paper is the use of the H2 boundary condition, which means an imposed local heat flux around the wetted perimeter (with a non-uniform temperature distribution at the solid walls).

This kind of thermal boundary condition can be useful when the solid walls are made using low thermal conductive solid materials. In these cases, it is important to pay attention to the increase in the wall temperature close to the corners of the cross section.

With the rhombic geometry considered in this work, by varying the angle it is possible to emphasize this local effect with a very large increase in the wall temperature near the closer corners.

The temperature gradients play an important role in many practical applications. In some cases, they must be controlled in order to verify that the wall temperature does not exceed the critical temperature for the specific wall material [44], while in other cases, they can be beneficial. In particular, temperature gradients play a leading role in the performance evaluation of Knudsen pumps. Operating this kind of micro-pump only requires a temperature gradient; therefore, Knudsen pumps are reliable and they do not require any maintenance, because they do not have moving parts [45]. In this context, the numerical outcomes of this work can be useful for designers and technicians involved in the sizing of Knudsen pumps.

## 2. Numerical Model

### 2.1. Mathematical Model

**Figure 1:**
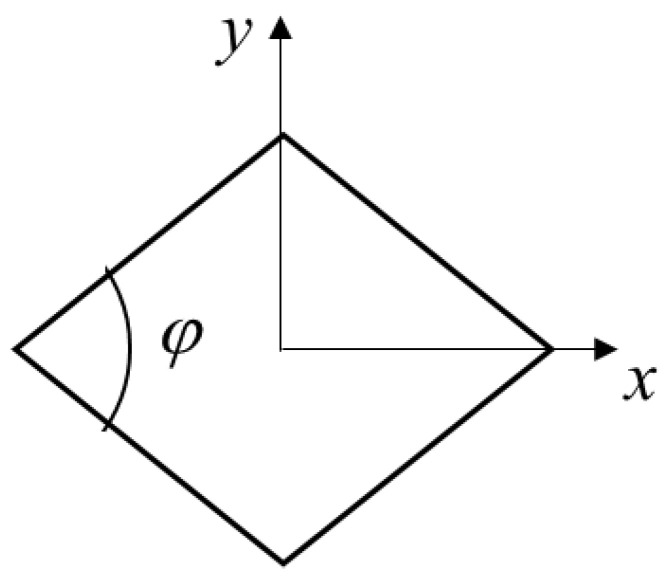
Sketch of the rhombic microchannel.

The microchannel investigated in the present study is shown in Figure 1, where *φ* represents the side angle. 

By assuming that the flow was laminar and fully developed and the fluid was Newtonian with constant physical properties, the momentum conservation equation was written as follows:

(1) $$\mu \left(\frac{\partial^{2}u}{{\partial x}^{2}}+\frac{\partial^{2}u}{{\partial y}^{2}}\right)=\frac{\partial p}{\partial z}$$

where *μ* is the fluid dynamic viscosity, *u* is the local velocity, and *∂p*/*∂z* is the pressure gradient. 

Because the analysis was carried out by assuming low values of the Mach number, compressibility was neglected [20]. 

The energy conservation equation was read as follows:

(2) $$\varrho c_{p}u\frac{\partial T}{\partial z}=\lambda \left(\frac{\partial^{2}T}{{\partial x}^{2}}+\frac{\partial^{2}T}{{\partial y}^{2}}\right)$$

where *ρ*, *c_p_*, and *λ* are the fluid density, specific heat at constant pressure, and thermal conductivity, respectively.

The temperature gradient along the axial direction of the microchannel was calculated from the energy balance equation for an elemental volume:

(3) $$\varrho c_{p}WA\frac{\partial T}{\partial z}=qP$$

where *W* is the average fluid velocity, *q* is the uniform wall heat flux, and *A* and *P* are the area and the perimeter of the cross-section, respectively.

The governing equations were rewritten in non-dimensional form by introducing the following dimensionless quantities:

(4) $$x^{*}=\frac{x}{D_{h}}\;\;\;\;\;\;\;\;\;\;\;\;\;\;\;y^{*}=\frac{y}{D_{h}}\;\;\;\;\;\;\;\;\;\;\;\;\;\;z^{*}=\frac{z}{D_{h}}$$

(5) $$u^{*}=\frac{u}{W}\;\;\;\;\;\;\;\;\;\;\;\;\;\;\;T^{*}=\frac{\lambda T}{{qD}_{h}}\;\;\;\;\;\;\;\;\;\;\;\;\;\;p^{*}=-\frac{D_{h}^{2}}{\mu W}\frac{\partial p}{\partial z}$$

The dimensionless governing equations are read as follows:

(6) $$\frac{\partial^{2}u^{*}}{{\partial x^{*}}^{2}}+\frac{\partial^{2}u^{*}}{{\partial y^{*}}^{2}}=p^{*}$$

(7) $$\frac{\partial^{2}T^{*}}{{\partial x^{*}}^{2}}+\frac{\partial^{2}T^{*}}{{\partial y^{*}}^{2}}={4u}^{*}$$

In the slip flow regime, the Navier–Stokes equations are solved subject to the velocity slip and temperature jump boundary conditions. To include the velocity slip at the channel walls, Equation (6) was solved by assuming the following boundary condition [1]:

(8) $$u^{*}-u_{w}^{*}=\frac{2-\sigma_{v}}{\sigma_{v}}Kn{\left(\frac{\partial u^{*}}{\partial n^{*}}\right)}_{w}$$

where *Kn* represents the Knudsen number.

The solution to Equation (7) was obtained by assuming the H2 boundary condition [43]:

(9) $${\left(\frac{\partial T^{*}}{\partial n^{*}}\right)}_{w}=-1$$

where n* was the outward normal vector. The heat transfer rate was evaluated by considering the Nusselt number averaged on the cross-section:

(10) $$Nu=\frac{hD_{h}}{\lambda }=\frac{1}{\bar{T_{w}^{*}}-T_{b}^{*}}$$

where $\bar{T_{w}^{*}}$ was the average dimensionless wall temperature and $T_{b}^{*}$ the dimensionless bulk temperature, which was calculated as follows:

(11) $$T_{b=}^{*}\frac{1}{A^{*}}\int_{A^{*}}^{}u^{*}T^{*}{dA}^{*}$$

where A* denoted the dimensionless cross-sectional area.

The dimensionless governing equations are modeled by using the partial differential equations module included in the commercial CFD code COMSOL Multiphysics^®^ 6.1. For the domain discretization, second-order elements for both the velocity and the temperature fields are considered, because this scheme works well for low flow velocities [46]. As a convergence criterion, a relative tolerance equal to 1 × 10^−6^ is imposed.

### 2.2. Grid Independence Analysis

The grid independence analysis was carried out by considering several mesh refinements. Due to the interest in the heat transfer rate, for each mesh refinement, the average Nusselt number (i.e., Equation (10)) was calculated, and the relative error *ε_Nu_* was evaluated as follows:

(12) $$\varepsilon_{Nu}=\left|\frac{Nu-{Nu}_{finest}}{{Nu}_{finest}}\right|$$

where *Nu_finest_* represented the Nusselt number obtained by adopting the finest mesh (i.e., a mesh characterized by at least 100,000 elements).

**Figure 2:**
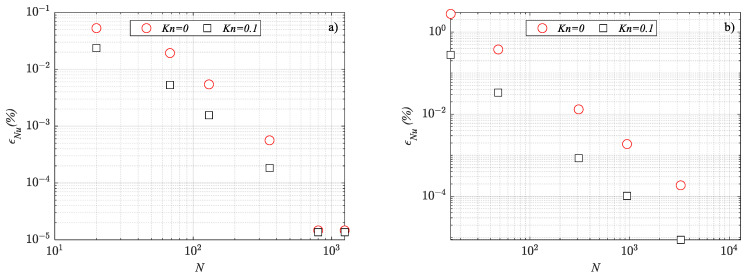
Results of the grid independence analysis: (**a**) *φ* = 10°; (**b**) *φ* = 90°.

**Figure 3:**
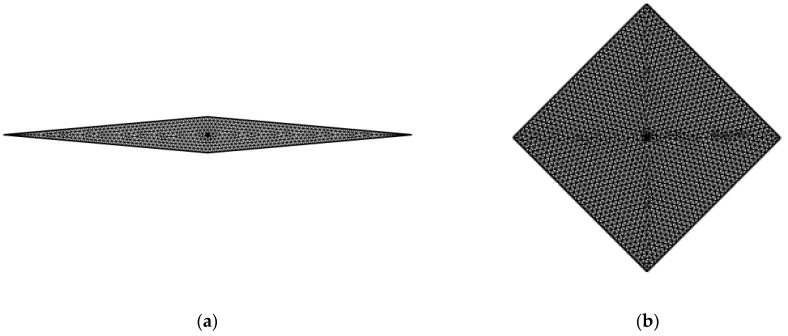
Adopted mesh: (**a**) *φ* = 10°; (**b**) *φ* = 90°.

In Figure 2, the results of the grid independence analysis are presented for the highest and the lowest side angles considered in the present study. It can be observed that the relative error *ε_Nu_* tends to zero when the number of elements becomes higher than 1 × 10^−3^, which means that a mesh-independent solution is achieved. Similar trends were observed by analyzing the remaining Knudsen number and side angle values. A sketch of the meshes adopted for the simulations is presented in Figure 3 for the same values of the side angle.

### 2.3. Model Validation

The accuracy of the numerical results was checked using the data available in the literature. In particular, for the no-slip flow, the numerical Nusselt numbers were compared to the results presented by Shah, who analyzed laminar forced convection heat transfer in ducts of arbitrary cross-section geometry by applying a least-squares-matching technique [42]. 

Good agreement was found for all values of the side angle considered in the present investigation, as indicated in Table 1. The maximum difference between the numerical values of the Nusselt number and the data reported in [42] is about 2.4% and occurs for the lowest value of the side angle considered here (i.e., *φ* = 10°).

**Figure 4:**
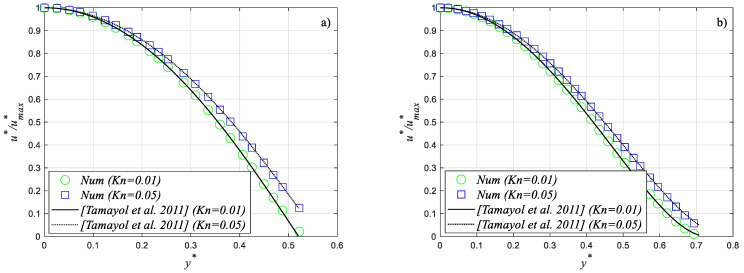
Comparison between numerical velocity profiles and analytical distributions [39] for two values of the Knudsen number: (**a**) *φ* = 33.5°; (**b**) *φ* = 90°.

To validate the numerical procedure in the slip flow regime as well, a comparison between numerical velocity profiles and the distributions obtained by applying closed form solutions for fully developed slip flow proposed by Tamayol and Hooman [39] was performed. A perfect agreement was found even in the slip flow regime, as shown in Figure 4. 

## 3. Results and Discussion

The analysis was carried out by considering several rarefaction degrees (i.e., Knudsen numbers) and different side angles of the rhombus. More specifically, the Knudsen number changed in the range 0–0.1, while the side angle was in the range 0.1–1, keeping the hydraulic diameter constant. 

The numerical results presented in this section were obtained by considering nitrogen as a working gas and by assuming a diffuse reflection and a perfect energy exchange [1].

**Figure 5:**
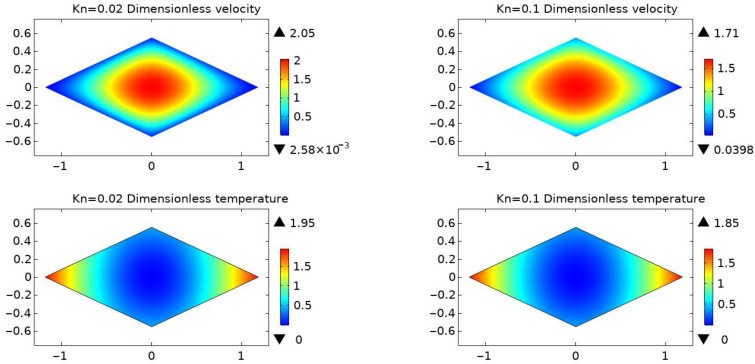
Dimensionless velocity and temperature contours for *φ* = 50°.

The dimensionless velocity and temperature distributions are presented in Figure 5 for *φ* = 0.5 and for two values of the Knudsen number; it can be observed that velocity slip at the wall increases with increasing *Kn*. Moreover, the rarefaction degree also affects the maximum velocity; in Figure 5, it can be observed that the maximum dimensionless velocity decreases as *Kn* increases. The same trend can also be observed for the dimensionless temperature.

The impact of rarefaction on fluid behavior depends on the value of the side angle. An increase or decrease in the Nusselt number was observed as the Knudsen number increased, depending on the value of the side angle. 

**Figure 6:**
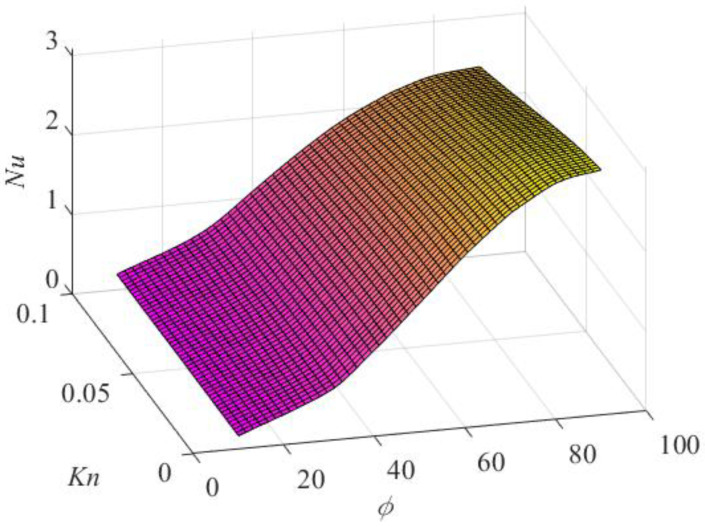
Nusselt number variation with the Knudsen number and side angle.

However, the numerical results revealed that *Nu* increased as the value of *φ* increased independently of the value of the Knudsen number, as shown in Figure 6, where the Nusselt number is presented as a function of both the Knudsen number and the side angle.

The influence of the side angle on the Nusselt number is more evident by considering the trend of the Nusselt number as a function of the Knudsen number for each value of the side angle. 

**Figure 7:**
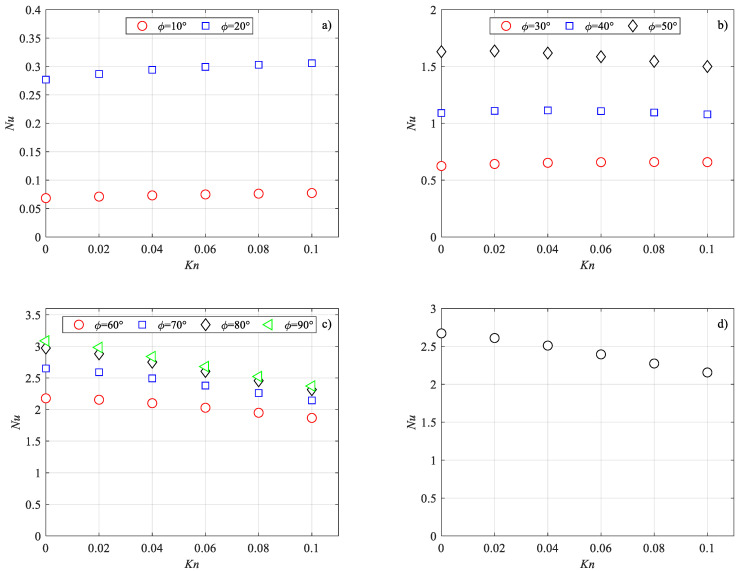
Nusselt number variation with the Knudsen number: (**a**) *φ* < 30°; (**b**) 30° ≤ *φ* ≤ 50°; (**c**) 60° ≤ *φ* ≤ 90°; (**d**) *φ* = 70.52°.

In Figure 7, this trend is presented for all values of the side angle considered here. It can be observed that the Nusselt number presents trends that are completely different depending on the value of the side angle. In particular, three different trends were observed: (i) for *φ* < 30° (Figure 7a), *Nu* increases with increasing *Kn*; (ii) for 30° ≤ *φ* ≤ 50° (Figure 7b), *Nu* has a maximum value for *Kn* in the range between 0.02 and 0.08; (iii) for 60° ≤ *φ* ≤ 90° (Figure 7c), *Nu* decreases with increasing *Kn*.

Similar trends were observed by van Rij et al. [27], by Kuddusi et al. [24,29], and Vocale et al. [33], who investigated the influence of rarefaction effects in rectangular, trapezoidal, and elliptic microchannels, respectively.

Since rhombic cross-sections can be obtained from the etching of silicon wafers, as discussed in the Introduction section, the influence of rarefaction effects on fluid behavior was also investigated for *φ* = 70.52°, which corresponds to a trapezoidal cross-section with an apex angle of 54.74° [20]. In Figure 7, the variation of the Nusselt number with the Knudsen number for *φ* = 70.52° is also presented. 

The trend of the Nusselt numbers for the rhombic cross-section with *φ* = 70.52° is similar to the trends observed by Kuddussi and Çetegen [29] in the fully developed flow region of trapezoidal microchannels characterized by different values of the aspect ratio.

With the aim of helping designers, hot and cold regions of the microchannels were investigated. According to Shah and London [42], the normalized maximum and minimum dimensionless wall temperatures were evaluated:

(13) $$t_{w,max}^{*}=\frac{T_{w,max}^{*}-T_{c}^{*}}{\bar{T_{w}^{*}}-T_{c}^{*}}\;,\;\;\;\;\;\;\;\;t_{w,min}^{*}=\frac{T_{w,min}^{*}-T_{c}^{*}}{\bar{T_{w}^{*}}-T_{c}^{*}}\;\;$$

where $T_{w,max}^{*}$ and $T_{w,min}^{*}$ represent the maximum and minimum dimensionless wall temperatures, respectively, and $T_{c}^{*}\;$ represents the dimensionless fluid temperature at the center of the rhombus, which in the present analysis was assumed to be equal to zero.

**Figure 8:**
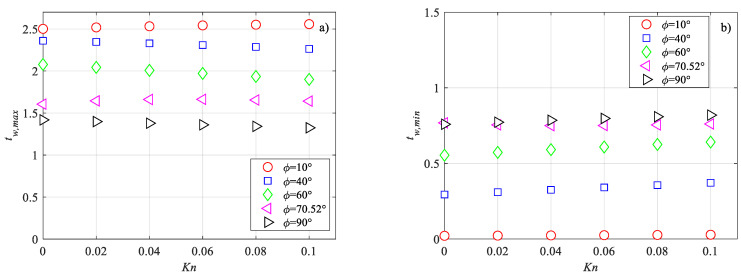
Variation of the dimensionless wall temperature with the Knudsen number: (**a**) maximum; (**b**) minimum.

In Figure 8, the trends of the normalized maximum and minimum dimensionless wall temperatures for some representative values of the side angle of the rhombus are presented. It can be observed that for low values of the side angle, the normalized maximum dimensionless wall temperature (Figure 8a) slightly increases with increasing Knudsen number, while for side angles higher than 20°, it decreases as the Knudsen number increases. The normalized minimum dimensionless wall temperature (Figure 8b) is a decreasing function of the Knudsen number, independently of the value of the side angle.

## 4. Conclusions

In this work, the heat transfer in rhombic microchannels under H2 boundary conditions was numerically investigated. A fully developed flow within the slip flow regime and under laminar steady state conditions was analyzed.

The numerical outcomes revealed that the rarefaction degree and the side angle strongly affected the Nusselt number. In particular, for low values of the side angle of the rhombus, rarefaction leads to an increase in the Nusselt number, while for high values of the side angle, rarefaction leads to a decrease in the Nusselt number.

The maximum convective heat transfer enhancement is about 13% and occurs for *φ* = 10°, while the maximum reduction in the convective heat transfer coefficient is about 23% and occurs for *φ* = 90°.

The results presented in this paper allow the designers of micro thermal devices to be able to take into account the effects linked to the non-uniform distribution of the wall temperature, which can be responsible for thermal deformations in the channels.

Under this perspective, the presented numerical analysis can be considered a source of data for designers and technicians involved, as an example, with the sizing of Knudsen pumps.

Moreover, the numerical results presented here highlight that the thermal performance of rhombic microchannels is interesting. Future works will be devoted to comparing rhombic, rectangular, trapezoidal, and triangular microchannels to evaluate which guarantees the best performance. Finally, the analysis will be extended to account for other effects, such as viscous dissipation and thermal creep.

## Figures and Tables

**Table 1:** Validation of the numerical model in a continuum flow regime.

*φ*	*Nu_num_*	*Nu* [42]
10	0.068	0.070
20	0.277	0.279
30	0.624	0.624
40	1.090	1.090
45	1.355	1.340
50	1.630	1.620
60	2.177	2.160
70	2.651	2.640
80	2.973	2.970
90	3.087	3.090

## Data Availability

Data are contained within the article.
